# Harms of harmless therapies

**DOI:** 10.1016/j.xfre.2024.02.001

**Published:** 2024-02-04

**Authors:** Richard J. Paulson

**Affiliations:** USC-HRC Fertility, Keck School of Medicine, University of Southern California, Los Angeles, California

In the past, the term “quackery” was commonly used in the context of the utilization and promotion of products that do not work or have not been proven to work (1, 2). Today, they’re called “supplements.” It is an unusual patient who comes to the office seeking fertility care that is *not* taking some form of supplement to help treat their infertility (3). Some patients even express a desire to delay their fertility treatment until they’ve had a chance to get back on supplements and make sure that they have achieved the correct level of whatever it is that they intend to ingest. Surprisingly, some fertility clinics actually include supplement recommendations in their information to patients. When queried, most of my colleagues shrug their shoulders. At worst, it seems, these supplements are harmless and patients like to feel they’re doing “everything they can” to help the treatment succeed. What harm is there from these “harmless therapies?”

This is a question that is worth addressing. Arguably, useless therapies have been a part of medical practice from the very beginning. The placebo effect is well understood. Why not afford a patient the opportunity to benefit (even if just psychologically) from a harmless placebo? It is worth considering the risks and benefits of supplements, because these types of unproven treatments are not entirely “harmless.”

In his 1999 editorial, Alistair MacLennan, the editor-in-chief of the journal, “Climacteric,” argued that there were at least 4 potential harms associated with the use of unproven therapies for the treatment of perimenopausal symptoms (4). His reasoning is still timely today and can be applied to the treatment of infertility. The first harm lies in the potential unanticipated side effects of drug interactions. In the context of fertility treatment, for example, there may be unknown interactions between the herbs and teas dispensed by acupuncturists and fertility medications, such as estrogen receptor modulators or ovarian stimulation medications. It may also be possible that supplements may interfere with hormonal assays, in a manner analogous to the way that biotin interferes with the measurement of thyroid stimulating hormone. The second harm is the high cost of ineffective treatments and the consequent waste of the healthcare dollar. It is worth asking our patients how much they pay for the supplements they buy; the costs are often quite high to inflate the perceived value of such alternative medicines. The third harm is the possibility that effective evidence-based treatment may be delayed. The biological clock is very real. The false strategy of delaying treatment while taking supplements for some period (such as 3 months) in an effort to “improve egg quality” can cause real harm. The fourth harm is subtle but real. When an unproven therapy fails to help, the patient experiences disappointment and disillusionment. One of the main reasons for the failure of in vitro fertilization is the cessation of treatment because of emotional exhaustion. We do not need to add to patient burn-out by offering, or worse, suggesting, multiple daily doses of unproven “harmless” therapies.

Most patients (and likely, most clinicians) are under the impression that dietary supplements and herbs are closely regulated to ensure that they are safe and effective. This is not the case. In 1994, Congress passed the Dietary Supplement Health and Education Act (DSHEA), which gave legitimacy to so-called “dietary supplements” (5). Through the DSHEA, even hormones like dehydroepiandrosterone and melatonin could be marketed as “supplements.” They are not subject to the same standards of quality control, nor are they proven to be effective. The only disclaimer that is required by the DSHEA on the label is: “This statement has not been evaluated by the FDA. This product is not intended to diagnose, treat, cure or prevent any disease.” The disclaimer is often overlooked, and when a supplement is actually recommended by the fertility physician or the clinic, likely ignored entirely.

Our field has already been overrun by so-called “add-ons,” tests and procedures with unproven benefits. Their incorporation into clinical practice generally follows a standard pattern. A reasonable hypothesis is followed by a small preliminary study which provides promising results. Without any confirmatory studies, the procedure or test is sold and immediately marketed. It then takes many years to complete the randomized clinical trials that disprove its usefulness. Even then, many clinicians are so accustomed to relying on the (useless) data provided by the test or the perceived effect of a useless procedure that they continue to use it. The incorporation of supplements into fertility treatment is even more insidious and harder to control. The internet is a wealth of medical misinformation, and anecdotal stories about unproven therapies abound. Social media posts may even be sponsored by producers of supplements. So-called “influencers” are not required to post disclaimers or to disclose their financial relationships with brands that they are promoting. Our patients are a highly susceptible audience, intimidated by the unnaturalness of fertility treatment, searching for some “natural” method of treating their infertility.

As clinicians and scientists, we need to push back on the antiscientific sentiment so prevalent in our culture today. We may begin by resurrecting the term, “quackery.” The alternative is to allow our patients to waste time and resources on unproven therapies, as well as tests and procedures. As Professor MacLellan wrote some 25 years ago (4), “At the end of the day, there should be no difference in the therapeutic regulations for a conventional or an unconventional medicine, therapy or so-called health food. For instance, an extract of yam that is sold as a health supplement but heavily promoted by third parties as having therapeutic actions should be subjected to the same trials and rigorous evaluation as an extract of yam promoted by a pharmaceutical company for the relief of menopausal symptoms.” Or, in the case of our subspecialty, for the treatment of infertility.

### CRediT Authorship Contribution Statement

**Richard J. Paulson:** Conceptualization, Writing – original draft, Writing – review & editing.

## Declaration of Interests

R.J.P. has nothing to disclose.
